# Group participants’ experiences of a patient-directed group-based education program for the management of type 2 diabetes mellitus

**DOI:** 10.1371/journal.pone.0177688

**Published:** 2017-05-16

**Authors:** Kate Odgers-Jewell, Elisabeth A. Isenring, Rae Thomas, Dianne P. Reidlinger

**Affiliations:** 1 Faculty of Health Sciences and Medicine, Bond University, Gold Coast, Queensland, Australia; 2 Centre for Research in Evidence-Based Practice, Bond University, Gold Coast, Queensland, Australia; University of Stirling, UNITED KINGDOM

## Abstract

**Objective:**

The objective of this study was to explore the experiences of individuals who participated in a group-based education program, including their motivators in relation to their diabetes management, and the perceived impact of group interactions on participants’ experiences and motivation for self-management. Understanding individuals diagnosed with diabetes experiences of group-based education for the management of type 2 diabetes mellitus may guide the development and facilitation of these programs.

**Methods:**

Semi-structured interviews were conducted with all individuals who participated in the intervention. Using thematic analysis underpinned by self-determination theory, we developed themes that explored participants’ motivators in relation to diabetes management and the impact of group interactions on their experiences and motivation.

**Results:**

The key themes included knowledge, experience, group interactions and motivation. Participants perceived that the group interactions facilitated further learning and increased motivation, achieved through normalization, peer identification or by talking with, and learning from the experience of others.

**Conclusions:**

The results support the use of patient-centred programs that prioritize group interactions over the didactic presentation of content, which may address relevant psychological needs of people diagnosed with type 2 diabetes mellitus, and improve their motivation and health behaviours. Future group-based education programs may benefit from the use of self-determination theory as a framework for intervention design to enhance participant motivation.

## Introduction

People with chronic diseases face many obstacles, including having to rely on a medical system largely designed for acute illness.[1] Chronic diseases pose distinctive challenges to our health care system, with sufferers requiring frequent, ongoing access to health services and medications, and often developing complex multi-morbidities.[2] For the most part, individuals with chronic disease generally manage their own condition, making up to 99% of their health-related decisions without input from formal health services.[3]

Patient education is the basis of effective chronic disease self-management and is essential to achieving improved outcomes for individuals with chronic disease.[4, 5] The goals of type 2 diabetes mellitus (T2DM) self-management education are to prevent complications, optimize quality of life and metabolic control, and reduce or prevent reliance on health care systems.[6] Research has shown that diabetes education leads to a range of outcomes including increased knowledge and understanding of diabetes, better self-management, heightened self-determination, enhanced psychological adjustment, and improved clinical outcomes.[7]

Group-based education programs offer many potential advantages over individual education. Group programs allow time for the provision of more detailed information, decrease time demands on health workers’ schedules, allow incorporation of families and carers into the education process, facilitate discussions and provide support from others facing similar challenges.[8] The benefits of group-based education for the management of T2DM, when compared with individual care alone, include significant benefits for clinical, lifestyle and psychosocial factors potentially substantially improving the outcomes of people with T2DM.[9–11] Additionally, research has shown that providing education in a group format rather than individually allows participants to explore their attitudes, and analyze their motives for current behaviours, potentially motivating them to improve their self-management skills and behaviours.[12] Group-based education programs therefore, may be more effective than individual education in empowering and motivating individuals to take responsibility for managing their condition.[12]

Self-determination theory [SDT] is a theoretical framework explaining the motivational dynamics affecting health behaviours.[13] It proposes that humans have three innate psychological needs that are the basis for their self-motivation and personality integration, and are essential for ongoing psychological growth, integrity and wellbeing: competence; relatedness; and autonomy. According to SDT, competence is feeling effective and exercising one’s capacities; relatedness is feeling respected, understood and cared for by others; and autonomy is the perception of being in charge of one’s own behaviour.[13, 14] Meeting these three needs may help to motivate the initiation and long-term maintenance of health-promoting behaviours.[13, 15] Unlike other theoretical frameworks, which focus on the quantity of motivation, SDT is more concerned with the type of motivation.[13] The use of SDT as a conceptual framework to study motivational processes has been supported by a recent systematic review.[14]

According to SDT, an individual’s motivation and behavioural regulation, or ability to act in accordance with their values, can be categorized as either ‘autonomous self-regulation’, ‘controlled regulation’, or ‘amotivation’.[13, 14] ‘Autonomous’ motivation is intrinsic and is based on the reflected endorsement in which people perceive that their behaviour emanates from themselves and find personal meaning from their behavioural consequences.[13, 14] In contrast, ‘controlled’ motivation is introjected and is externally regulated by pressure to meet demands or obtain rewards,[13, 14] while ‘amotivation’ refers to a state of lacking any intention to act.[13, 14] The more autonomously motivated individuals are, the more adaptive their behaviour potentially resulting in improvements in health outcomes.[14, 16]

To understand individuals’ experiences of group-based education for the management of T2DM, and to guide the development and facilitation of these programs in the future, this research aimed to explore the experiences of individuals who participated in a group-based education program.

The theoretical framework of SDT was used to explore two research questions:

1. What are group participants’ motivators in relation to their diabetes management?
2. What impact do participants perceive that group interactions have on their experiences and motivation for self-management?

## Methods

We used qualitative data obtained from semi-structured interviews with the participants of a group-based education program for the management of T2DM to explore their experiences of the program. The intervention is described in detail elsewhere.[17] Briefly, the intervention was a patient-centred, patient-directed, group-based education program for the management of T2DM. The intervention was developed using data from a preliminary literature review, a formative evaluation of interviews with the facilitators and participants from a range of chronic disease management group education programs, and a review of the Medicare group services information pack available to Australian health professionals.[18, 19] The program was evaluated using both quantitative measures to assess the effectiveness of the intervention, and qualitative interviews to assess the acceptability of the intervention. The intervention resulted in improvements in quantitative outcomes, and was acceptable to participants.[17] After program completion, telephone interviews were conducted with participants by a researcher independent to the program.

Previous content analysis of the interview data formed a process evaluation, which allowed the researchers to explore group participants’ preferences for group program structure and facilitation, their satisfaction with the program and their outcomes. The current study was a secondary analysis of the interview transcripts, which allowed the researchers to obtain a deeper understanding of group participants’ experiences, motivators, and the effect of the group interactions on their motivation to self-manage their T2DM through the lens of SDT. Secondary analysis of qualitative data explores research questions different from those asked in the primary data analysis. This enables researchers to disentangle data from earlier perspectives and permit new findings to emerge.[20] In this way, secondary analysis can utilize descriptively rich qualitative data sets potentially leading to a deeper understanding of the data.[20]

### Data collection

Ethics approval was obtained from the Bond University Human Research Ethics Committee (Protocol Number RO1815) and verbal and written consent was obtained from the participants prior to the commencement of the intervention. Additionally, participants provided verbal consent prior to the commencement of the telephone interviews. Participants were invited to the group-based education program if they self-reported a diagnosis of T2DM or were referred by their GP as a diagnosed T2DM patient, were 18 years of age or over, had adequate cognitive ability, and had a sufficient understanding of English. Group participants were all recruited through feature stories in a free local newspaper. Thirty-three potential participants made initial contact with the researcher (KOJ) of which a total of 16 participants enrolled in the study. Three did not complete the intervention. Thirteen intervention participants agreed to take part in the telephone interviews, which represented the entire sample of intervention participants who attended and completed the six-week program.

Interview questions (Table 1) were developed prior to intervention commencement and were based on a previously developed interview schedule, which focused on a range of chronic disease management programs. The questions were further refined and piloted prior to intervention recruitment. The interviews were conducted by a dietitian external to the study with previous semi-structured interview experience. Prior to data collection, two pilot telephone interviews were undertaken within the research team. The interviews were audio-recorded, transcribed verbatim, checked, anonymized and corrected against the audio files by the first author (KOJ). No incentives were provided to group participants to take part in the intervention or telephone interviews.

**Table 1:** Interview schedule and inquiry logic for semi-structured interviews.

Objective:	Question:	Example Prompts:
**To explore individuals’ motivation and reasons for attending the program**	Why did you get involved in the program?	What was it about the program that attracted you to get involved?
**To identify individuals’ preferences for group program structure (number of contact hours, facilitator/s)**	Can you describe what you liked most about the program?	Was there anything specific that you particularly enjoyed?
	Can you describe what you liked least about the program?	Would you change anything about the program?
	What do you think the ideal program length would be (i.e. number of weeks, number of hours per week)?	Did you feel that six weeks was a good length, or would you like the program to be longer or shorter?
**To identify the effect of the group environment on the individuals learning and impression of support**	Please describe how the other people in the group helped or hindered your learning?	Did it help you at all to know that others in the group were in the same situation as you?
	How do you feel the group has contributed to any changes that you have made?	How did others in the group help you to make the changes you have made?
	What was the role of the group facilitator in your discussions within the group?	How did the group facilitator educate the group?
**To identify outcomes (confidence, self-efficacy, lifestyle changes, attitudes, health and knowledge of T2DM)**	Has your knowledge of type 2 diabetes changed since you started the program? How?	In terms of your knowledge, what kind of things do you feel you have learnt?
	How has your diet or exercise changed since you started the program?	Is your diet the same as before you started the program? What has changed?
	How has your blood glucose testing changed since starting the program?	How often were you testing before starting the program? How often do you test now?
	How have your diabetes control and your confidence in managing your diabetes changed since starting the program?	How do you feel you are managing your diabetes since starting the program?
	How have your health and attitudes changed since you started the program?	How is your attitude towards diabetes different since starting the program?
**To explore individuals’ satisfaction with the program.**	Would you recommend this program to your friends?	Why or why not?

Note: Interview questions were used as a guide and may have slightly differed between participants

### Data analysis

Two authors (KOJ & DPR) completed an initial thematic analysis using an iterative approach including independent analysis followed by frequent discussions until agreement was reached on a final set of codes. The same two authors (KOJ & DPR) then identified preliminary themes and subthemes. Themes and subthemes were then mapped to the three key needs described in the SDT framework as overarching categories (Competence, Relatedness and Autonomy).[21]

The themes were analyzed using a hybrid deductive and inductive thematic analysis approach based on the pre-selected SDT.[21, 22] An inductive approach directly draws codes, categories, or themes from the data, whilst a deductive approach uses preconceived codes or categories derived from relevant theory, research, or literature.[23, 24] The deductive analysis allowed the use of a predetermined theory to enable an in depth exploration in line with a previously described social phenomenology, whilst the inductive analysis allowed themes to emerge directly from the data.[22]

One author (KOJ) wrote a summary of the themes and subthemes and identified illustrative quotations. A conceptual map was developed to illustrate the categories, themes and subthemes and their inter-relationships, which was discussed with the second researcher (DPR) to ensure integrity in the final presentation of results. The quotes presented in the results illustrate and exemplify the themes described.

## Results

The characteristics of the participants are presented in Table 2. The majority of participants were retired, aged 65 years or older, educated to a secondary school level, married, diagnosed 4 to 6 years ago and had never attended another group education program. Just over half of the participants were male. The intervention participants were predominantly Australian; however, some participants were born overseas.

**Table 2:** Characteristics of participant sample.

Attribute		N = 13
**Gender:**	Male	7
	Female	6
**Age:**	55–64 yrs	3
	65–74 yrs	5
	≥75 yrs	5
**Marital Status:**	Married	8
	Divorced	2
	Separated	1
	Widowed	2
**Education level:**	Primary	1
	Secondary	6
	Tertiary	6
**Employment status:**	Temporary	1
	Self-employed	1
	Retired	11
**Years since diagnosis:**	≤1 yr	2
	1–3 yrs	2
	4–6 yrs	4
	7–9 yrs	2
	≥10 yrs	3
**Previous group attendance:**	No	11
	Yes	2

The three needs proposed by SDT—competence, relatedness and autonomy—were used as the overarching categories in this analysis. Additionally, themes and subthemes identified during the process of data analysis reflected the breadth and depth of the concepts brought forward in the interviews (Table 3). Representative quotes from participants illustrating key response subthemes are presented in Table 4.

**Table 3:** Summary of SDT categories, themes and subthemes developed from the secondary analysis of telephone interview data.

Category	Theme	Subtheme
A. Competence	A1: Knowledge	A1-1 Change in knowledge
		A1-2 Facilitator as expert
		A1-3 Diet and behaviours; exercise and exercise knowledge
		A1-4 Confidence and diabetes control
	A2: Experience	A2-1 Time since diagnosis
		A2-2 Peer as expert
		A2-3 Self-monitoring of blood glucose testing improved
B. Relatedness	B1: Group Interactions	B1-1 Normalisation
		B1-2 Altruism
		B1-3 Facilitator support
		B1-4 Comparison with others
		B1-5 Peer support
		B1-6 Social aspect
		B1-7 Reassurance
		B1-8 Group discussions
		B1-9 Additional contact time
C. Autonomy	C1: Motivation	C1-1 Extrinsic
		C1-1-1 Motivated by others
		C2-1 Intrinsic
		C2-1-1 Interest
		C2-1-2 Seeking knowledge
		C2-1-3 Motivation for self-management
		C3-1 Amotivation
		C3-1-1 Lack of responsibility

**Table 4:** Representative quotes from participants illustrating key response subthemes developed from the secondary analysis of telephone interview data.

Subtheme	Example quote
A1-1 Change in knowledge	*You know*, *I learnt a bit about myself*, *it’s a good reminder of everything*, *what you should do*, *what you shouldn’t do*, *what to eat*, *what not to eat*. [Participant 3]
A1-2 Facilitator as expert	*I think facilitating the comments of people*, *making people feel comfortable to discuss anything that they are having a problem with… she was the oil in the whole thing she made it happen quite effortlessly*. [Participant 1]
A1-3 Diet and behaviours; exercise and exercise knowledge	*I am trying to eat healthy*, *trying to not have too much carbohydrate*, *and certainly try and cut down on the sugars wherever possible*. *I’m on a stationary bike*, *which I’m working on getting more and more on*, *but it’s very hard to get into exercise*. [Participant 4]
A1-4 Confidence and diabetes control	*I’ve really kept on*, *really just how I have been before actually going on the program*, *and I think like anything it just makes you more aware*. [Participant 10]
A2-1 Time since diagnosis	*A couple of people were knowledgeable*, *where they’d been doing it for a very long time*, *… a lot of it was probably old hat to them*, *and you know when you’ve been doing it more than ten years or longer… when someone raised a question*, *they were able to speak with experience and say well I’ve had that*, *I’ve been doing this for years and years*, *and this is the best way*. *There are certain things that [the group facilitator] wouldn’t have known probably*. [Participant 9]
A2-2 Peer as expert	*But I think that one particular fellow helped*, *I learnt more I would say off him than I did any of the others around me…*. *Some of them actually surprised me that*, *you know like one of the fellows there had been diabetic for a while*, *and knew next to nothing*, *I don’t think he even knew how to handle his needle properly*. [Participant 11]
A2-3 Self-monitoring of blood glucose testing improved	*I didn’t test before the program*. *I am testing now*. *I take one first thing in the morning*, *and then I try and take one two hours after breakfast*. [Participant 4]
B1-1 Normalisation	*So it was an environment among people who all probably had similar experiences*, *and that was quite good*. *I didn’t feel*, *like for example*, *should you tell other people who are non-diabetic or don’t know about it*, *they just think*, *oh yeah*, *have a look at other people*, *you look healthy*, *what’s wrong with you*, *you are a whinger*, *you know that is really the problem… you don’t want to go somewhere and say oh no I am a diabetic and I feel so bad*. [Participant 8]
B1-2 Altruism	*I thought… someone’s calling for volunteer type things to do with diabetes and I read it*, *…*. *and then I thought about it*, *… and I thought well I should ring and just see if I’m the type of person they’re looking for*. [Participant 9]
B1-3 Facilitator support	*[The facilitator] was just a delight*, *the way she ran it*, *the way she handled it*, *made it very easy to want to go back to the next week*, *you know rather than saying this is a bit of a bore I’ll give it a miss… We realised she was making a super effort… and it made it worthwhile to go*. [Participant 1]
B1-4 Comparison with others	*Well I think some of them were just*, *I could have been one of them*, *but are totally out of it*, *they have no idea about diet*, *… in fact I’m terribly worried about one or two of them*, *I’m sure they didn’t even do what I was hoping they’d do*. *I think it helped because I was not alone as being a total idiot*. [Participant 2]
B1-5 Peer support	*So it was all fairly simple*, *and very relaxed*, *because everybody could talk*, *everybody could say their thing*, *and everybody’s input to me was important*. [Participant 7]
B1-6 Social aspect	*Well*, *I found going there every Thursday*, *it was great*, *it was good companionship… the people were happy*, *I was looking forward to going*, *it was something to do*, *you know*, *of a Thursday*, *and I sort of missed it for a couple of Thursdays but it’s okay now*. [Participant 7]
B1-7 Reassurance	*I was aware that I had to do some exercise*, *so I was already in progress of doing the exercise*. *So*, *but it*, *you know*, *it just rubber-stamps it that that’s what I’ve got to continue doing*. [Participant 1]
B1-8 Group discussions	*The main thing was I listened to others*. *I hadn’t spoken to anyone else really with it*, *since I got it*, *to know how other people think*. [Participant 9]
B1-9 Additional contact time	*I could have found other things that could have been talked about*. *Ah*, *you could probably say maybe 10 [sessions]*, *depending on the sort of period of time*, *and of course it depends on people’s circumstances*, *what they’ve got to do*. [Participant 10]
C1-1 Extrinsic	
C1-1-1 Motivated by others	*It was motivating actually*, *really motivating*, *because it made me realise that if he’s on injections and he keeps as well as he does*, *and he wasn’t real young… and as fit as what he is*, *it most certainly was motivating that you can you know do that yourself*. [Participant 11]
C2-1 Intrinsic	
C2-1-1 Interest	*Because I would like to go ahead and… keep my health problems under control as I did so far for the past seven years actually*. *And I did that mainly*, *well I tried to at least*, *mainly with diet*, *my exercise approach is not too successful*, *I could do much more there*, *but I think it’s a good fresh up*. [Participant 8]
C2-1-2 Seeking knowledge	*I said well*, *I’ll give it a go to get more information and to learn a bit more what’s going on*. [Participant 7]
C2-1-3 Motivation for self-management	*Basically*, *because I have diabetes*, *and if I can learn something more about it*, *or about what I can do for myself*, *then I’ve gained*. [Participant 12]
C3-1 Amotivation	
C3-1-1 Lack of responsibility	*To be quite truthful*, *I still don’t think about my diet*, *I have to pull myself up*, *you know like… I went for morning tea the other day*, *… I sat down*, *I had… sandwiches I had cakes*, *you name it*, *and then said to the girl I was with*, *I’m going to have problems tonight*, *it’s going to be my own fault*, *and I wasn’t even thinking the sugar*. [Participant 11]

**Fig 1:**
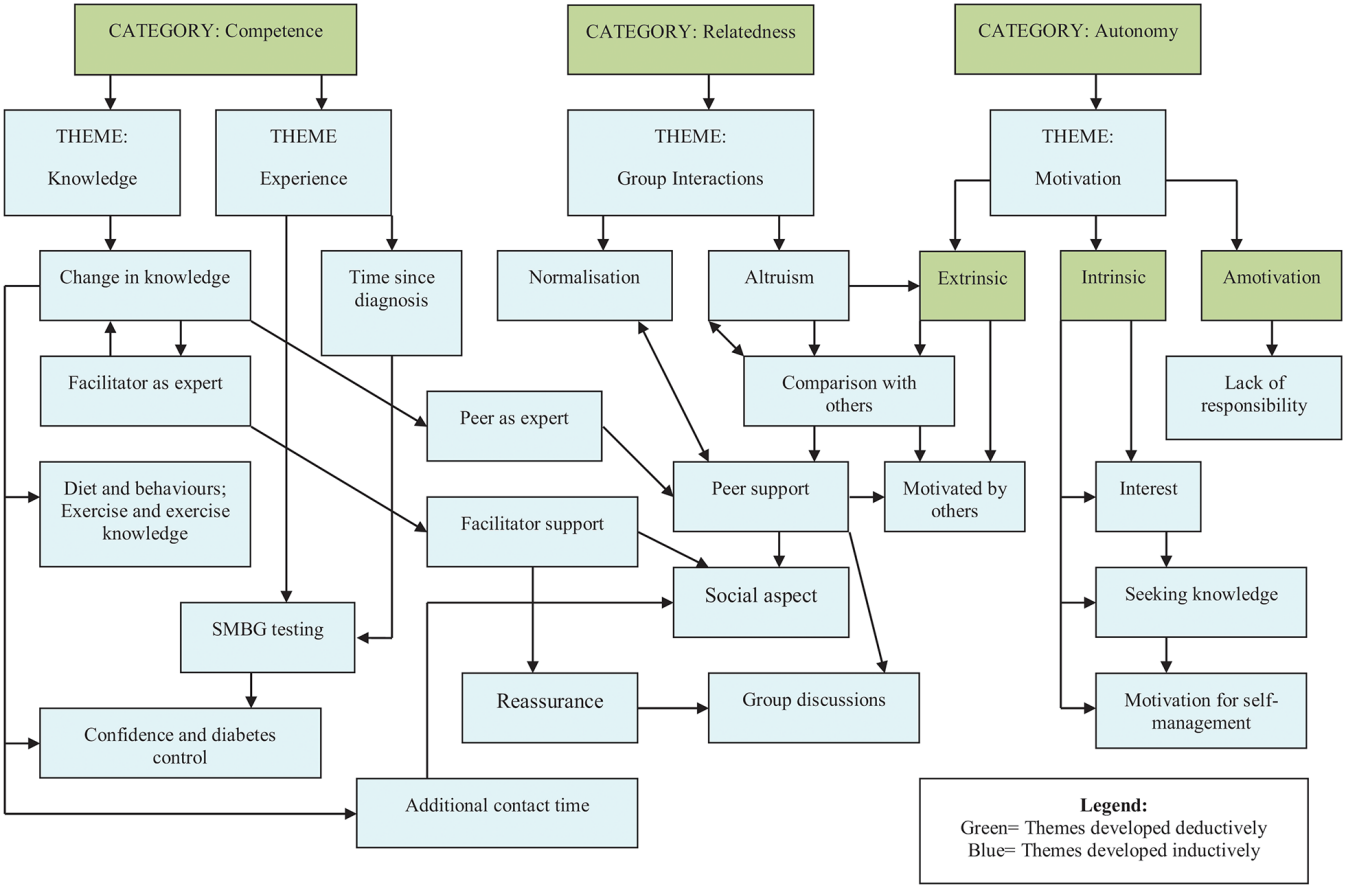
Conceptual map of themes developed related to group participants’ experiences of the intervention.

Themes and subthemes are presented in a conceptual map (Fig 1). During the analysis, the researchers perceived these themes and subthemes to often be linked and inter-related, and these interrelationships are represented with arrows in Fig 1. Thematic inter-relatedness suggests that enhancing one aspect of an individual’s self-determination may enhance other aspects, such as their motivation.

### SDT: Competence

Competence was organized into two themes, Knowledge and Experience. The desire to gain or improve knowledge was a clear motivator for all participants, and appeared the prime motivator to attend the group-based education program. Within this theme, participants spoke of their change in knowledge related to T2DM due to the intervention, with only one of the participants stating that his knowledge remained unchanged. Increased knowledge was described in three main areas, diet, exercise, and self-monitoring of blood glucose (SMBG). Improvements in knowledge were generally attributed to either the group facilitators’ knowledge, or the knowledge of other participants. Participants perceived to place great value on experiential knowledge.

Participants described identifying more experienced peers and respecting their opinions and knowledge over others in the group. Participants commonly associated time since diagnosis and experience of T2DM with increased knowledge and self-management skills. At times, participants reported being surprised that experienced participants lacked knowledge and self-management skills, as they assumed that time since diagnosis was associated with improvements in these areas.

A majority of participants claimed to have made changes in their behaviours as a result of the knowledge gained from the group-based intervention, including changes in diet, exercise, SMBG testing, diabetes control and confidence. The only participant who did not report any physical changes in his behaviours was the most experienced participant. However, he did report being more aware of his diet, exercise and diabetes management.

### SDT: Relatedness

Relatedness captured participants’ experience of group-based education. There was one key theme, Group Interactions. This theme encompassed various subthemes including normalization, altruism, facilitator support, comparison with others, peer support, social aspects, reassurance, and group discussions. These were often interrelated, and included interactions between other group participants, and with the group facilitator. A key subtheme, normalization, captured participants’ realization that other participants had situations similar to their own. Some of the male participants, who had been diagnosed for a number of years, noted that they had never spoken to anyone about their diabetes before coming to the group, but felt comfortable to share their thoughts, concerns and questions within the program.

Normalization was closely linked to another subtheme, comparison with others. All of the participants described comparing themselves to others in the group, whether negatively or positively. Comparing themselves to others tended to either motivate them to improve or reassure them that they were doing well. Reassurance was also related to Competence. When comparing to those seen as ‘doing better’ than themselves, participants were either motivated to improve or looked up to these peers as experts. In contrast, when comparing to those seen as ‘doing worse’ than themselves, participants felt reassured, appeared more confident, or were concerned and wanted to help those they perceived were faring worse. Some participants noted that they were able to obtain some perspective by seeing others who seemed to not be coping, whilst some considered themselves to be different from others because of the specifics of their situation (e.g. one unmedicated participant stated that she was different as she was diet-controlled).

Peer support was also important to participants. Most participants noted that their peers in the group had provided support to them in various ways. They attributed this to other group members listening to their stories or questions, sharing personal information, having group discussions, and relating with them on a social level. Facilitator support also appeared to motivate some participants. For example, the facilitator taking interest in them in various ways, such as making them feel welcome and comfortable, listening to their stories, answering their questions, demonstrating respect, being open and friendly, and including them in discussions.

The majority of the participants reported enjoying the social aspect of the group-based intervention, possibly because most of the participants were retired and may have lacked regular social interaction. Providing participants with morning tea in each session allowed them to move around the room and have conversations with others in the group, encouraging the social aspect of the program. Some participants reported being reassured during the group-based intervention, mainly from the facilitator, however, at times by peers or by comparison with others.

A subtheme related to both Competence and Relatedness was additional contact time. Some participants mentioned that they would have liked the program to go for longer, whilst others were happy with the amount of contact time. Those wanting the program to be extended generally felt that more contact time would allow more time for group discussions and socializing, and believed that this may improve competence. Some participants did realize that others had commitments outside of the program, and that increasing the contact time may make participants less likely to commit to lengthy group programs.

An interesting subtheme that emerged was that of altruism (helping others). Many participants reported an altruistic motivation to participate in the program, however some appeared to want to participate in the program in order to improve their own self-esteem. The majority of the participants who discussed helping others were referring to other people with T2DM, however one participant referred to helping his children should they be diagnosed down the track.

### SDT: Autonomy

In relation to an individual’s perceived ability to self-manage their condition the key theme was Motivation. Some participants described various motivators, categorized as either extrinsic or intrinsic. Other participants were categorized as ‘amotivated’ in accordance with the predetermined SDT category, as they were perceived to lack the intention to self-manage their condition.

Extrinsic factors that motivated participants to learn about and improve their diabetes self-management included comparison with and motivation from others. These were often linked. For example, participants who compared themselves with others and felt that others were better managed than themselves seemed motivated to improve their own management. Most of the participants described intrinsic motivators to attend the intervention including being motivated out of interest, knowledge seeking or an internal desire to improve their self-management. Those participants motivated by knowledge seeking or interest usually had some knowledge but felt they needed a refresher, or had minimal knowledge and were not coping well with their diabetes. A few newly diagnosed participants’ interview responses indicated ‘amotivation’ or described what seemed to be a lack of intention to act or change their self-management behaviours. Some described rationalizations such as sugar cravings, the weather affecting their ability to exercise, looking for miracle cures, unfounded views and a false sense of security.

## Discussion

Using SDT as an analytic framework, qualitative telephone interviews of participants in a T2DM group-based program explored participants’ experiences of the program, their motivators in relation to their diabetes management, and the impact of group interactions on their experiences. Three categories (Competence, Relatedness and Autonomy) encompassed the developed themes of Knowledge, Experience, Group Interactions and Motivation.

Knowledge and Experience were two subthemes of Competence. Similar to previous research (where group participants valued the opportunity to gain additional knowledge and report improvements in knowledge [25]), participants highlighted knowledge seeking as a motivator for attending the program. Participants additionally expressed a desire to gain knowledge and improve competence from the intervention to improve their self-management activities, such as meal planning, medication administration, regular physical activity, and home glucose monitoring.[8] Adopting self-management skills is necessary to enable people with T2DM to effectively manage their diabetes,[26] and successful self-management requires sufficient knowledge of the condition and its treatment.[27] Participant self-report suggests that the intervention was successful in improving knowledge and consequently competence, with participants reporting various behaviour changes such as improvements in diet, exercise and exercise knowledge, and SMBG.

Participants attributed their improvements in knowledge to both the facilitator and peers. Peers in a group situation can offer knowledge, practical skills, personal competence, emotional support, and provide encouragement beyond the capacity of many health professionals.[28] Furthermore, participants considered that peers who had been diagnosed for longer than them as more knowledgeable. This insight suggests that it may be helpful to include more experienced peers in group-based education programs to improve the knowledge and competence of individuals newly diagnosed with T2DM. The WHO has recognized peer-support programs as a valuable and promising approach to diabetes education and management.[8] Previous research has identified the important role of the facilitator in setting the tone and guiding the direction of groups, which may significantly influence the participant outcomes.[29]

Feelings of relatedness (feeling understood, respected and cared for by others [13, 14]) was experienced through group interactions. Participants expressed that others in the group positively influenced them to learn and achieve changes in various areas of their diabetes management via peer identification, learning from other’s experiences, and feeling inspired by role models or motivated by those who were experiencing complications that they wanted to avoid. Group interactions and peer identification have been shown to improve individuals with T2DM self-esteem, self-perception and self-efficacy, and to promote awareness, empowerment, and positive attitudes towards diabetes.[30] Social support provided by strangers, has been linked to improvements in self-management, psychological functioning and biomedical outcomes,[31] and identified as a clinically relevant factor on the pathway to glycaemic control in people with T2DM.[32] Utilizing a patient-directed approach, in which the content of the program is decided by the participants, therefore reflecting participants’ own needs and questions, may encourage group discussions and group interactions. Previous research has indicated that when utilizing a patient-directed approach, participants pay close attention to the information provided, were motivated to make the changes they selected, attrition may have been improved, and participants were able to discuss their experiences, concerns and questions which resulted in lively and relevant sessions.[33]

Autonomy as it relates to SDT, explored the motivators of group participants and interview data were themed to align with extrinsic motivation, intrinsic motivation or ‘amotivation’. Extrinsic (external) motivators identified in the data included being motivated by others or motivated by comparing oneself with others. Intrinsic (internal) motivators identified included being motivated by interest, knowledge seeking, or an internal desire to improve self-management behaviours. Intrinsically motivated individuals are more likely to experience improved behaviours and health outcomes.[13] These participants could be considered empowered. Empowerment is a concept used to describe individuals’ acceptance of responsibility to manage their own condition and solve their own problems using information, rather than directives, from health professionals.[34] Patient empowerment literature views internal motivation as a more effective motivator for lifestyle change than external motivation, as at times individuals are externally motivated to make changes only to please their health professional, not usually resulting in long-term change.[34]

‘Amotivation’ refers to the state of lacking any intention to act.[13, 14] A few newly diagnosed participants’ interview responses indicated ‘amotivation’ or a perceived lack of intention to act in order to improve their health and self-management. Other research has also reported that some individuals newly diagnosed with T2DM lack the intention to manage their condition,[25, 35] and tend to only take ownership of their diabetes and seek out more specific or detailed information once they have reached a degree of acceptance of their disease.[36] When receiving a diagnosis of diabetes, people are faced with new challenges and behaviours that are unknown and therefore they may lack the perception of competence or the feeling of being effective in their own management.[14]

### Strengths and limitations

Qualitative interviews were an ideal method to explore participants’ experiences and perspectives of the intervention. Qualitative methods can provide rich and diverse data that are not obtainable through quantitative means.[37] Additionally, research has shown that obtaining participants’ perspectives on group-based education can reflect individuals’ real-life experiences and potentially result in data rich in human experience.[12]

Data trustworthiness was achieved by independent analyses of the data by two authors (KOJ & DPR). Themes and subthemes were discussed until agreement was reached ensuring that the analysis was credible, and that no common themes or subthemes were missed.

Semi-structured interviews, primarily constructed of open-ended questions and probes, allowed group participants to provide in-depth information, which may have been missed using other research methods. However, the use of semi-structured interviews may have influenced participants’ responses by prompting them to talk about topics that they may not have discussed otherwise. The interviews were conducted by a third party rather than the group facilitator in order to reduce the potential impacts of a perceived power differential and participants’ potential reservations to be honest and comprehensive in their responses, particularly in relation to the group facilitator.

An additional strength of the study was the inclusion of individuals from a range of backgrounds with variations in the years since diagnosis. The majority of participants were aged over 65 years, which is likely due to the facilitation of the group program on weekday mornings when retired persons could attend. There is likely to have been some sampling bias—the sample characteristics of the group participants were dissimilar to the characteristics of participants in the AusDiab study.[38] Health professionals interested in particular sub-groups of the population not represented in the sample may wish to consider specific research with the community of interest.

All intervention participants agreed to take part in the telephone interviews, reducing any potential sampling bias, however the sample size was small due to recruitment difficulties. Although all participants were represented, the limited sample size makes it difficult to ascertain whether theoretical saturation was achieved. Research has shown that theoretical saturation is obtainable using six to twelve participants with interviews as the mode of data collection.[39] For the purpose of the qualitative component of this group-based education study, sample representativeness was not necessary, as the researcher was exploring lived experiences of individuals with T2DM in a real world setting. As with most qualitative research the results of this study should not be generalized beyond this group of participants or beyond the particular intervention.

A potential source of participant bias was that only participants who completed the course were invited to take part in the interviews. Alternate views may have been offered by those who elected not to take part in the intervention or did not complete the whole program. Additionally, it is possible that those who volunteered to participate in the intervention may have been more motivated than the average person with T2DM, which may have resulted in improved outcomes in comparison to ‘amotivated’ individuals.

## Conclusions

This qualitative study is the first to demonstrate the application of the SDT to group-based education for the management of T2DM when viewed from the perspective of the participants themselves. A clear benefit of group-based education for the management of chronic diseases is the impact of relatedness.[21] Unlike individual education, group-based education provides direct opportunities for people to learn from peers, to be supported by peers, to compare themselves with others in the same situation, to socialize and to feel as though they have helped others. Relatedness seems to have impacted the motivation of individuals in the group, which aligns with the premise of the SDT that relatedness is one of the psychological needs that is the basis of self-motivation.[13, 21] Additionally, the enhanced effectiveness of patient-directed and patient-centred interventions may be considered through the lens of the SDT, which suggests that improving individuals’ competence by encouraging relatedness and the feeling of autonomy improves their motivation and health behaviours.[13, 21] Previous research has shown that treating individuals with T2DM as autonomous and equal contributes to patient satisfaction.[40]

In conclusion, the themes generated in the secondary analysis of the qualitative interviews align with SDT, suggesting that group-based education programs that foster group interactions may be addressing relevant psychological needs of individuals with T2DM and could improve their motivation. Previous research has shown that meeting the innate needs identified by SDT can motivate individuals to initiate and maintain health behaviours over the long-term.[13, 15] Group-based education programs appear to provide a critical forum for relatedness. Future group-based education programs may benefit from the use of SDT as a framework for intervention design to enhance participant motivation.
